# Variation in apoptosis mechanisms employed by malaria parasites: the roles of inducers, dose dependence and parasite stages

**DOI:** 10.1186/1475-2875-11-297

**Published:** 2012-08-28

**Authors:** Holly Matthews, Medhat Ali, Victoria Carter, Ann Underhill, Jennifer Hunt, Hannah Szor, Hilary Hurd

**Affiliations:** 1 Centre for Applied Entomology and Parasitology, Institute for Science and Technology in Medicine, School of Life Sciences, Keele University, Keele, Staffordshire, ST5 5BG, UK; 2 School of Environment and Life Sciences, College of Science and Technology, University of Salford, Salford, Greater Manchester, M5 4WT, UK; 3 Department of Zoology, Faculty of Science, Ain Shams University, P.BOX 11566, Cairo, Egypt

**Keywords:** Apoptosis, Malaria, *Plasmodium berghei*, Ookinetes, Chloroquine, Reactive oxygen species, Density

## Abstract

**Background:**

*Plasmodium berghei* ookinetes exhibit an apoptotic phenotype when developing within the mosquito midgut lumen or when cultured *in vitro*. Markers of apoptosis increase when they are exposed to nitric oxide or reactive oxygen species but high concentrations of hydrogen peroxide cause death without observable signs of apoptosis. Chloroquine and other drugs have been used to induce apoptosis in erythrocytic stages of *Plasmodium falciparum* and to formulate a putative pathway involving cysteine protease activation and mitochondrial membrane permeabilization; initiated, at least in the case of chloroquine, after its accumulation in the digestive vacuole causes leakage of the vacuole contents. The lack of a digestive vacuole in ookinetes prompted the investigation of the effect of chloroquine and staurosporine on this stage of the life cycle. Finally, the suggestion that apoptosis may have evolved as a strategy employed by ookinetes to increase the fitness of surviving parasites was explored by determining whether increasing the ecological triggers parasite density and nutrient depletion induced apoptosis.

**Methods:**

Ookinetes were grown in culture then either exposed to hydrogen peroxide, chloroquine or staurosporine, or incubated at different densities and in different media. The proportion of ookinetes displaying positive markers for apoptosis in treated samples was compared with controls and results were analyzed using analysis of variance followed by a Turkey’s test, or a Kruskal-Wallis test as appropriate.

**Results:**

Hydrogen peroxide below 50 μM triggered apoptosis but cell membranes were rapidly compromised by higher concentrations, and the mode of death could not be defined. Both chloroquine and staurosporine cause a significant increase in ookinetes with condensed chromatin, caspase-like activity and, in the case of chloroquine, phosphatidylserine translocation and DNA fragmentation (not investigated for staurosporine). However, mitochondrial membrane potential remained intact. No relationship between ookinete density and apoptosis was detected but nutrient depletion significantly increased the proportion of ookinetes with chromatin condensation in four hours.

**Conclusions:**

It is proposed that both a mitochondrial and an amitochondrial apoptotic pathway may be involved, dependent upon the trigger that induces apoptosis, and that pathways may differ between erythrocytic stages and ookinetes, or between rodent and human malaria parasites.

## Background

Since Picot and colleagues first suggested that the crisis forms of cultured intra-erythrocytic stages of *Plasmodium falciparum* were undergoing apoptosis [1], acceptance of the putative existence of this form of programmed cell death, and discussion of the role it may be playing in the life history of the malaria parasite, has been growing [2-4]. A number of studies now report observations of morphological and biochemical markers of apoptosis (recently reviewed in [5]). A few are contradictory, but it has been proposed that problems surrounding the use of differing parasite strains, induction with different drugs and drug doses and variation in life cycle stages, including sequential intra-erythrocytic stages, confound the ability to evaluate and compare studies [5]. Almost all reports of an apoptosis-like phenotype (hitherto referred to as apoptosis) in *Plasmodium* spp. have been performed *in vitro* on intra-erythrocytic stages of *P. falciparum*. However, detailed studies have also been made, both *in vitro* and *in vivo*, of ookinetes of the rodent malaria, *Plasmodium berghei*[3,6-8] and it was becoming clear that a more systematic examination of apoptosis inducers and markers needed to be made in an attempt to link reports concerning these different species and stages together. This was the aim of this study.

Classical biochemical markers, including loss of mitochondrial membrane potential (ΔΨ_m_), DNA fragmentation and the activation of caspase-like proteases, have been observed in both species [6,9-11]. Although alterations in the plasma membrane, such as externalization of phosphatidyl serine, cannot be detected in intra-erythrocytic stages of *P. falciparum* due to the various membranes surrounding the parasite, they have been reported in extracellular ookinetes (discussed in [12]). Caspase-like activity has been detected in both intra-erythrocytic and ookinete stages but the nature of these caspases is unclear as, in common with other parasitic protozoan’s that exhibit markers of apoptosis [12], the *Plasmodium* genome does not contain classical caspases. However, both clan CD metacaspases and clan CA cysteine proteases have been implicated in *P. falciparum* apoptotic pathways [10,11,13,14]. Recently genes orthologous to other components of apoptotic pathways in metazoan apoptosis have been identified using bioinformatics, but, so far, only in the *P. falciparum* genome. In addition to metacaspases, homologues of Tudor Staphylococcal Nuclease, ZEN1, a putative apoptotic DNase and AIF (apoptosis inducing factor) have been identified [15,16]. *Plasmodium falciparum* apoptosis-related protein (*Pf* ARP) has been purified and localized to the cytosol but no role in apoptosis has yet been defined [17]. However, disruption of the ClpQY protease machinery located in the mitochondria led to loss of ΔΨ_m_ and, one to two hours later, activation of a caspase-like cysteine proteases and DNA fragmentation [18].

The majority of studies of *P. falciparum* have used drug treatment to induce apoptosis [1,9,19-21], whereas studies of *P. berghei* ookinetes have been conducted without the application of toxic drugs [6,7]. Triggers that occur naturally within the mosquito midgut, which have been shown to induce apoptosis in *P. berghei* ookinetes, include nitric oxide (NO) and reactive oxygen species (ROS) [8]. The ROS generator 3, 4-dihydroxy-L-phenylalanine (L-DOPA) acts in a dose- and time-dependent manner, whereas hydrogen peroxide (H_2_O_2)_ caused death without detectable features of apoptosis. Death was defined as significant loss of cell membrane integrity. It occurred after one hour of exposure, even at the lowest concentration tested (100 μM) [8]. The conflicting results obtained when different donors of ROS were used in an attempt to induce ookinetes to undergo apoptosis may have been due to enhanced sensitivity to H_2_O_2._ This explanation has been tested here by exposing ookinetes to lower doses of H_2_O_2_ than had been used previously.

Inhibition of the activity of NO synthase in the mosquito significantly reduced the number of ookinetes with activated caspase-like molecules in their cytoplasm at 15 hr post-infection and increased the intensity of oocyst infection 12 days post-infection. However, although *in vitro* incubation with the NO donor sodium Nitroprusside resulted in a significant increase in nuclear chromatin condensation, PS translocation and caspase-like activity, it did not induce the loss of ΔΨ_m_, indicating that there may be more than one apoptotic pathway operating in ookinetes [8].

Using micromolar concentrations of chloroquine (CQ) and staurosporine (ST) to induce several features of apoptosis in *P. falciparum* trophozoite stages, Ch’ng and co-workers [10,22] have begun to delineate a putative cell death pathway in this species and life cycle stage. They suggest that, following CQ exposure, activation of cysteine proteases results in mitochondrial outer membrane permeabilization (MOMP). Importantly, following accumulation of CQ in the digestive vacuole, destabilization of the membrane was recognized as the initial downstream event that released Ca^2+^ and cysteine proteases into the parasite cytosol [22]. They propose that both this damage to the diges-tive vacuole, the resultant MOMP and subsequent amplification of cytoplasmic cysteine protease activity resulted in DNA fragmentation and death and conclude that cysteine proteases are acting both up- and down-stream of MOMP. Inhibitor studies suggested the cysteine proteases belonged to clan CA, rather than CD [10].

There are several discrepancies between observations of apoptosis features pertaining to putative pathways in ookinetes of *P. berghei* and those of the trophozoite stages of *P. falciparum* outlined above. In *P. berghei*, apoptosis can be inhibited by cyclohexamine (suggesting some part of the process relies on protein synthesis) [6] and also by clan CD cysteine protease inhibitors, including the general caspase inhibitors, Z-VAD.fmk and boc-ASP as well as the more specific caspase-3 inhibitor, Z-DEVD.fmk [6], but not by clan CA cysteine protease inhibitors E64d and K11777 [23]. These differences may result, in part, from differences in morphology. Notably, although ookinetes contain haemazoin crystals as a legacy from macro gametocytes, they do not feed by using a phagosome and lack a digestive vacuole that could accumulate CQ or initiate the pathway that has been proposed for intra-erythrocytic stages. As an initial step towards ascertaining whether multiple PCD pathways may exist in different *Plasmodium* spp., and/or at different stages in the life cycle, ookinetes were exposed to CQ and to ST and monitored for several markers of apoptosis to determine whether these drugs initiated apoptosis.

It is becoming clear that there are several triggers that induce features of apoptosis in *Plasmodium*. In addition to drug treatments, NO derivatives and oxidative stress [8,20], high parasite density has been implicated [11]. In order for density to be a trigger that initiates apoptosis in protozoan parasites, a mechanism to sense the presence of conspecifics must be present. It has been suggested that protozoan parasites form communities in which individuals may communicate with each other in a manner similar to the quorum sensing seen in bacteria [16,24,25]. At times of nutrient stress this communication may be advantageous if it results in the altruistic suicide of some parasites, thus reducing competition for nutrients or limiting host/vector damage [26]. Indeed, nutrient stress itself may be an inducer of apoptosis and this concept has been explored here. As well as exposure to drug treatment, ookinetes were cultured at different densities and in different nutrient conditions so that the effect of parasite density and nutrient depletion could be investigated. Thus, the objective of this study was to further the understanding of the process of apoptotic cell death in malaria parasites by integrating recent reports on the process in intra-erythrocytic stages with observations made on the extracellular ookinete stage. Although CQ and ST were also found to act as inducers of apoptosis in ookinetes, the effect of chemical and ecological triggers on various morphological and biochemical markers support the view that more than one apoptotic pathway may exist in ookinetes.

## Methods

Products were purchased from Sigma Aldridge Company Ltd (Dorset, England, UK) unless otherwise stated. All animals were handled in strict accordance with good animal practice as defined by the UK Animal (Scientific Procedures) Act 1986 and approved by the UK Home Office, license number PPL 40/2997. Work was also approved by the University of Keele Animal Care and Ethical Review Committee. All incubations with reagents were carried out at 19°C (a temperature appropriate for *P. berghei* ookinetes).

### Parasite culture and harvesting

*Plasmodium berghei* ANKA 2.34 was maintained by passage in Charles River-derived mice (CD1). Blood containing exflagellating gametocytes collected by cardiac puncture and then incubated in RPMI 1640 medium, supplemented with foetal bovine serum, sodium bicarbonate, hypoxanthine and antibiotics (ookinete medium), for 18–22 hr to allow for the formation of ookinetes [27]. To harvest ookinetes from this culture, erythrocytes were lyses by incubation on ice with 0.17 M ammonium chloride (1:50 vol/vol) for 20–30 min. The remnants of the red blood cells were removed by washing twice with cold phosphate buffered saline (PBS). Ookinetes were pelleted by centrifugation at 800 *g* for 5 min and resuspended in 1 ml PBS or ookinete medium for counting.

### Apoptosis inducers

In order to determine whether the anti-malarial drug CQ and the apoptosis inducer ST act as triggers of apoptosis in the ookinete stage, as well as the intra-erythrocytic stage, parasites were incubated in the presence of these drugs. Chloroquine diphosphate was dissolved to a concentration of 1 mM in RPMI or ookinete medium. Solutions were prepared fresh and stored in the dark prior to use. Two different protocols were used to test the effect of CQ on ookinetes. Initially, a range of dilutions was prepared from the stock CQ/RPMI solution and a 200 μl aliquot of ookinetes, resuspended in PBS, was added to 1 ml of each dilution and incubated for 2 hr. Controls were incubated in RPMI medium alone. Thereafter, ookinetes were resuspended directly in 1 ml of 1 mM chloroquine/ookinete medium and incubated for 2 hr. Controls for each replicate were incubated in ookinete medium alone.

Staurosporine (ST) was dissolved in 1 ml dimethylsulphoxide (DMSO) to make a 2.14 mM stock solution and stored at 2-8°C. The stock solution (7 μl) was added directly to 1 ml of ookinetes suspended in ookinete medium to give a final concentration of 15 μM and ookinetes were incubated in 1 ml of this for 30 min. Preliminary experiments had established that DMSO at this concentration did not cause ookinetes to die and informed the dose and incubation time used in this experiment (unpublished data). Ookinetes incubated in ookinete medium alone served as controls for each replicate experiment.

Hydrogen peroxide was diluted to 1 mM with PBS and stored for no more than 1 week. RPMI 1640 was used to prepare a range of concentrations from this stock solution and 1 ml added to ookinetes resuspended in 200 μl PBS and incubated for 1 hr. For each replicate experiment, controls were incubated in ookinete medium only.

Following treatment with each inducer, ookinetes were washed two to three times with cold PBS and immediately assayed for apoptotic activity.

### The effect of ookinete density and incubation medium on the proportion of ookinetes undergoing apoptosis

To determine whether the ecological triggers, high parasite density or nutrient depletion, acted to induce apoptosis, ookinetes were washed, counted and incubated further for 4 hr in RPMI medium supplemented with 10% foetal bovine serum, RPMI medium alone or PBS, at densities of 1, 2 and 4 x 10^7^ ookinetes/ml. Ookinetes were immediately assayed for signs of condensed chromatin (at least two replicates of counts of 50 ookinetes) and experiments were repeated three times. In a further experiment, ookinetes were incubated in PBS for 4, 6 and 24 hr before assaying for condensed chromatin.

### Markers of apoptosis

Following treatment, ookinetes were assayed for morphological markers of apoptosis using techniques adapted from those used for metazoans. Ookinetes stained with fluorochrome markers of apoptosis were viewed under oil immersion (x1,000) using a Leica DM IRB inverted microscope. Low parasite yield and time constraints restricted the use of all the markers in some experiments.

Chromatin condensation was detected using acridine orange, a fluorescent cationic dye that stains nuclear material. Three μl of acridine orange (final concentration 2.5 μg/ml PBS) was added to ookinetes suspended in an equal volume of PBS on a microscope slide. Vectashield mounting medium (0.5 μl) (Vector Laboratories Inc) was added and the samples were viewed immediately. Live ookinetes stained uniformly green, nuclei with condensed chromatin were stained an intensified green.

An annexin V-FITC Apoptosis Detection Kit was used to detect translocation of phosphatidylserine (PS) to the external leaflet of the plasma membrane according to the manufacturer’s instructions, except that incubations were performed for 12 min followed by the addition of 1 μl of propidium iodide (PI), followed by a further 3 min incubation. Ookinetes were mounted with Vectashield (0.5 μl) containing 4’, 6-diamidino-2-phenylindole (DAPI) (Vector Laboratories Inc) prior to examination. Ookinetes staining PS positive/PI negative were regarded as apoptotic.

The activation of caspase-like molecules was detected using CaspaTag^TM^ Pan-Caspase *In Situ* Assay Kit (Chemicon International, USA). The assay is based on fluorochrome inhibitors of caspases (FLICA) that bind covalently to activated caspases. This kit contains the carboxyfluorescein-labelled fluoromethyl ketone peptide inhibitor of caspases (FAM-VAD-FMK). The assay was carried out following the manufacturer’s instructions except that 250 μM PI was used in some of these assays to detect cells with compromised membranes. Samples were mounted with Vectashield or Vectashield containing DAPI or PI (as indicated in the results) (Vector Laboratories Inc) and viewed immediately. Staining of activated caspases was taken as a marker for apoptotic cells. PI staining is indicative of a compromised membrane which could be due to necrotic cell death or a late stage of apoptotic cell death.

Collapse of ΔΨ_m_ resulting in mitochondrial outer membrane permeabilization (MOMP) was detected using the JC-1 assay kit (Invitrogen) according to manufacturer’s instructions. This is regarded as a reliable fluorescent probe for use with intact cells [28] and has been used with appropriate controls in previous studies as a marker of apoptosis [7]. Samples were mounted with Vectashield containing DAPI prior to examination. In live cells, the presence of a negative charge established by an intact mitochondrial membrane potential allows the cationic, lipophilic dye to accumulate in the mitochondria, forming orange JC-1 aggregates. Mitochondrial depolarization and loss of mitochondrial membrane potential is indicated by loss of these aggregates leaving only green fluorescent staining throughout the cytoplasm.

The *In Situ* Death Detection Kit, Fluorescein (Roche) was used to detect DNA fragmentation. During apoptosis DNA strands break leaving the strand termini exposed. In this reaction the enzyme terminal deoxynucleotidyl transferase is used to catalyze the addition of fluorescently labeled nucleotides to free 3’-OH termini (TUNEL-reaction). The TUNEL assay was completed in accordance with the manufacturer’s instructions with a few modifications. Briefly, ookinetes were fixed onto positively charged SuperfrostPlus slides (Thermo Scientific) using neutral buffered 10% formalin solution, washed twice with PBS and stored at −20°C until further processing. Once thawed, ookinetes were permeabilized for 5 min at room temperature using 40 mM sodium citrate and 1% Titron X-100 (BDH Chemicals). One slide, which served as the positive control, was then incubated with 3U/ml DNAase 1 recombinant for 10 min to induce the breakage of DNA strands. All slides, apart from the negative control, were incubated with the TUNEL reaction mixture (freshly prepared) at 37°C for 75 min. The negative control was incubated with labeling solution only. Subsequent to staining, the slides were washed with PBS, air dried and mounted in Vectashield containing DAPI. In this study, ookinetes with stained nuclei were considered apoptotic whilst ookinetes with unstained nuclei were recorded as live. Although it is recognized that DNA fragmentation can occur during the process of necrosis, this assay was used in conjunction with other markers of apoptosis to confirm the apoptotic phenotype.

### Data analysis

Data were analyzed using Minitab® software (version 16) or Microsoft excel. The Anderson-Darling test was used to test for normality and normally distributed data analyzed using one- way or two-way ANOVA with a general linear model for two parameters. When significant differences were found, this was followed by a Turkey’s test for paired comparisons. If data were not normally distributed they were arcsine transformed prior to performance of a one-way ANOVA. If the arcsine-transformed data were not normal they were log transformed prior to analysis. If data was not normal after both data transformations, a non-parametric, Kruskal-Wallis test was carried out on the original data.

## Results

### The dose dependent effect of chloroquine on the induction of apoptosis in *Plasmodium berghei* ookinetes

**Figure 1:**
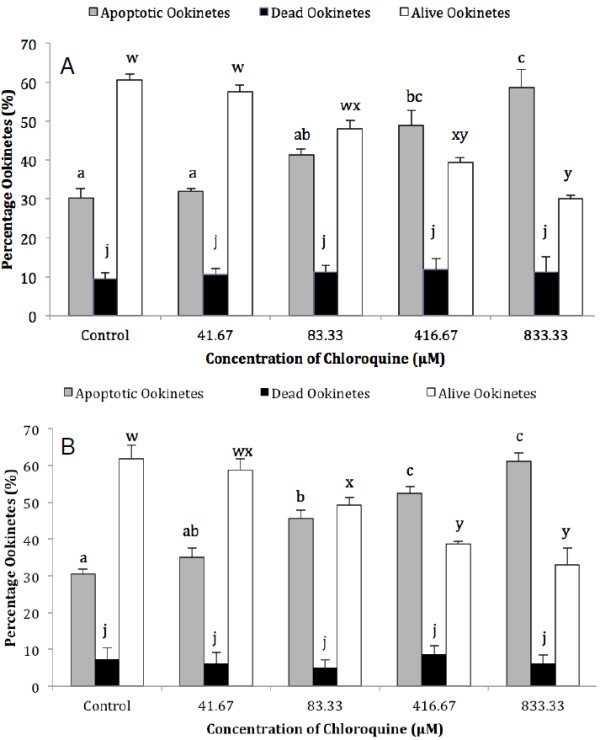
**The effect of chloroquine dose on *Plasmodium berghei *ookinetes *in vitro.*** Ookinetes were incubated for 2 h in a range of concentrations of chloroquine dissolved in RPMI 1640 medium. The proportion of parasites containing nuclei with condensed chromatin, detected using acridine orange staining (**A**), or expressing phosphatidyl serine on the outer surface, detected with annexin (**B**), was recorded as apoptotic. Staining with propidium iodide was used to identify dead ookinetes. Within a particular condition, bars that do not share the same letter are significantly different (*p*<0.05), n = 40 ookinetes per sample, error bars = SEM for four experimental repeats.

Following previous reports that CQ induced features of apoptosis in the erythrocytic stages of *P. falciparum* when parasites were exposed to micromolar concentrations of the drug [10], the effect of incubating *P. berghei* ookinetes in a range of final CQ concentrations from 41.67-833.33 μM (see Figure 1) was investigated. Assays for nuclear condensation and the translocation of PS to the outer surface of the cell membrane were used to detect signs that ookinetes were dying by apoptosis. The initial experiment was repeated four times and 40 ookinetes were observed for each of the two assays. Ookinetes that were dead or in the late stages of apoptosis were detected by the influx of PI into the cell. Live ookinetes were negative for PI (compromised membrane) and for acridine orange (condensed chromatin) or annexin staining (PS translocation) respectively. Following a 2-hr incubation, the proportion of ookinetes containing nuclei with condensed chromatin increased steadily with the dose of CQ and at 833.33 μM was almost double that in control samples (58.6% and 30.1% respectively, F_4,14_ = 15.23, *P* = 0.0001). Paired comparisons showed that this increase was significantly different from the controls at 416.67 μM CQ (Figure 1A). Similarly, translocation of PS to the external leaflet of the plasma membrane was induced by CQ (F_4,15_ = 35.43, *P* = 0.0001), and was again significantly different from the control samples at 416.67 μM CQ and above (Figure 1B). Notably, different concentrations of CQ induced a similar proportion of ookinetes to exhibit chromatin condensation or annexin staining respectively. No increase in the proportion of dead ookinetes was detected in either assay (Figure 1).

**Figure 2:**
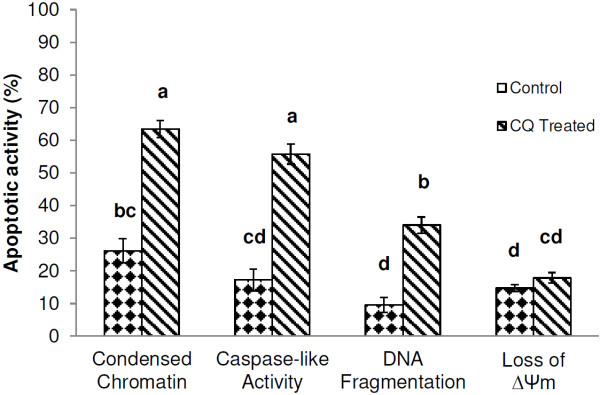
**The induction of apoptosis in *Plasmodium berghei *ookinetes following exposure to chloroquine.** Ookinetes were maintained in ookinete culture medium only (control) or treated with 1 mM chloroquine for 2 h (chloroquine treated). Following incubation, ookinetes were examined for the presence of four apoptotic markers: condensed chromatin, caspase-like activity, DNA fragmentation and loss of ΔΨ_m_ using the acridine orange, CaspaTag, TUNEL and JC-1 assays respectively. Error bars = SEM for three experimental repeats, n = 50–100 ookinetes per sample. Different letters indicate that data are significantly different.

To investigate further phenotypic and biochemical markers of apoptosis, ookinetes were incubated for 2 hr with 1 mM CQ dissolved in ookinete medium. In addition to chromatin condensation, three other apoptosis characteristics that had previously been observed in *P. berghei* ookinetes [7], namely: DNA fragmentation, caspase-like activity and loss of mitochondrial membrane potential were monitored. Overall, significantly more ookinetes exhibited markers of apoptosis than untreated ones (F_7,38_ = 56.72, *p>*0.001). Chloroquine again induced a significant rise in the proportion of parasites with condensed chromatin (26.14% to 63.43% *P* <0.05) that was a remarkably similar proportion to that produced by the highest dose (833.3 μM) in the previous experiment. Chloroquine also caused a significant increase in the number of ookinetes with an activated caspase-like cysteine protease (17.2% to 55.8% *P*<0.05) (Figure 2). Fewer untreated ookinetes had fragmented DNA, but this proportion also rose significantly upon treatment (9.5% to 34%, *P<*0.05, Figure 2). The increase in ookinetes displaying these apoptotic markers was not accompanied by a concomitant increase in loss of ΔΨ_m_, suggesting that CQ induced apoptosis via a mitochondrial-independent pathway during these experiments. In assays where PI was used to detect ookinetes with compromised membranes, CQ did not induce a significant increase in PI + ve ookinetes. The proportion of ookinetes exhibiting different markers at the same time post-incubation (and age) descended from condensed chromatin to caspase-like activity to DNA-fragmentation.

### Staurosporine induces markers of apoptosis in *Plasmodium berghei* ookinetes

**Figure 3:**
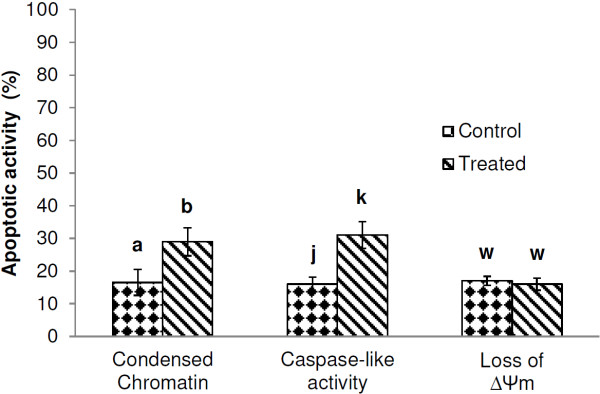
**The effect of staursoporine on *Plasmodium berghei *ookinetes.** Ookinetes were incubated in ookinete medium only (control) or 15 μM staurosporine for 30 min (staursoporine treated), washed with phosphate buffered saline and assayed for the presence of condensed chromatin, (acridine orange assay), caspase-like activity (CaspaTag assay), and loss of mitochondrial membrane potential (JC-1 assay). Paired T-tests were performed to compare the level of apoptotic activity for control and treated samples within each assay. Error bars = SEM for three experimental repeats, n = 50-100 ookinetes per sample. Within assays, significant differences are indicated by different letters.

Staurosporine has been shown to cause a significant increase in CaspaTag and TUNEL positive *P. falciparum* trophozoites and to decrease the proportion with a functional ΔΨ_m_[10]. *Plasmodium berghei* ookinetes were also found to be susceptible to ST in this investigation. Following a 30 min incubation there was a significant increase in ookinetes with condensed chromatin (*P =* 0.032*)* and caspase-like activity (*P =* 0.027) but, as with CQ, the JC-1 assay did not detect a significant loss of ΔΨ_m_ in the samples (*P =* 0.205*)* (Figure 3). The low yield of parasites obtained when these experiments were conducted precluded the investigation of other markers.

### The dose-dependent effect of hydrogen peroxide on markers of apoptosis in *Plasmodium berghei* ookinetes

**Figure 4:**
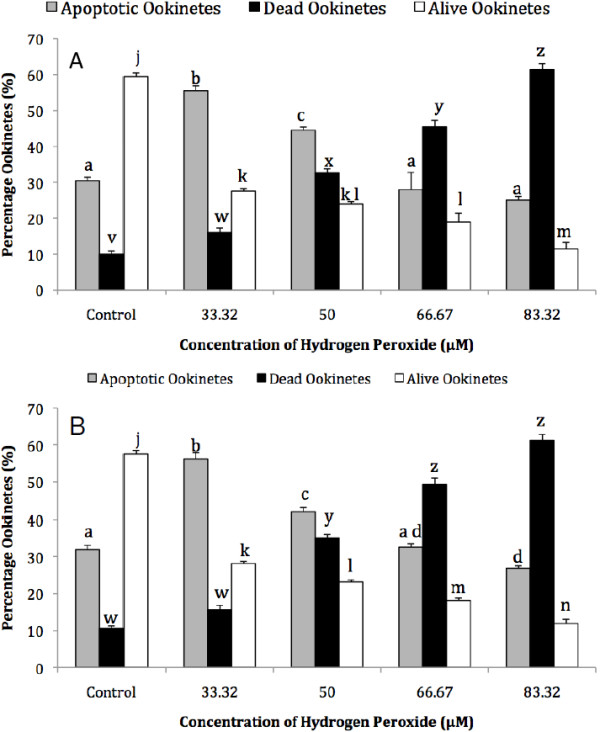
**The dose-dependent effect of hydrogen peroxide on the expression of markers of apoptosis in *Plasmodium berghei *ookinetes.** Ookinetes were incubated in a range of concentrations of H_2_O_2_/RPMI 1640 medium for 1 h. The proportion of parasites containing nuclei with condensed chromatin, detected using acridine orange staining (**A**), or expressing phosphatidylserine on the outer surface, detected with annexin (**B**), was recorded as apoptotic. Staining with propidium iodide was used to identify dead ookinetes. Within a particular condition, bars that do not share the same letter are significantly different (*p*<0.05), n = 40 ookinetes per sample. Error bars = SEM for four experimental repeats.

Previous work demonstrated that oxygen radicals, produced by a short incubation period with 100 or 500 μM L-DOPA, caused a significant increase in the proportion of ookinetes with condensed chromatin, whereas longer incubation periods or higher concentrations caused loss of membrane integrity [8]. These results may be due to the rapid onset of late apoptotic features or to necrotic cell death. In contrast, no increase in parasites with condensed chromatin occurred when they were incubated in similar concentrations of hydrogen peroxide, but those with loss of membrane integrity increased significantly, even with 100 μM H_2_O_2_. Here, lower concentrations of H_2_O_2_ were used and this showed that, compared with the control, there is a significant increase in ookinetes with two markers synonymous with early stage apoptosis; condensed chromatin and PS translocation, when incubated for 1 hr with 33.32 or 50 μM H_2_O_2_ (*P* = 0.0001 in each case) (Figure 4). The proportion of live ookinetes continued to decrease with increasing dose but there was a greater number of parasites with compromised membranes than those showing signs of early apoptosis at 66.67 and 83.32 μM H_2_O_2_.

### The effect of incubation medium and density on the induction of apoptosis in *Plasmodium berghei* ookinetes

**Figure 5:**
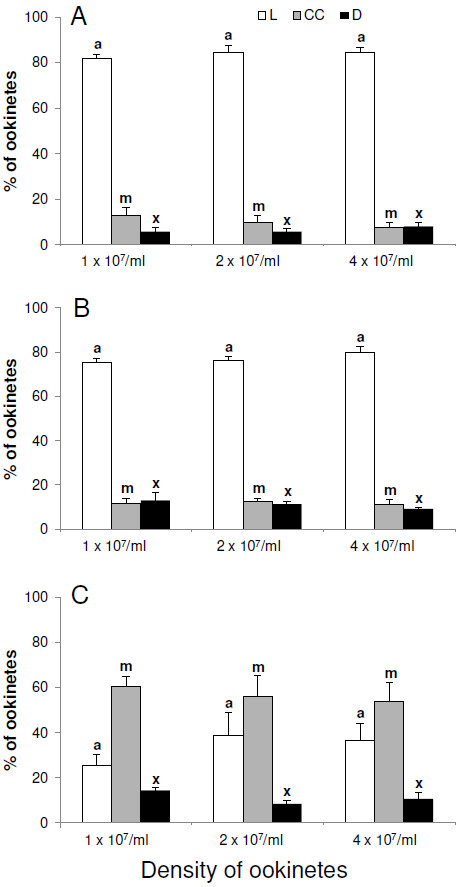
**The effect of incubation media and parasite density on nuclear condensation in *Plasmodium berghei. *** Following harvesting, ookinetes were incubated for 4 h in RPMI supplemented with; FBS (**A**), RPMI alone (**B**) or PBS (**C**). In each case three densities of ookinetes were used. Ookinetes showing chromatin condensation were identified by using acridine orange and classed as apoptotic (CC), those with a compromised membrane were detected with propidium iodide and classed as dead (**D**) and those with neither of these markers were alive (L). Error bars = SEM for three experimental repeats, each with at least two replicates of counts and each count = 50 ookinetes. The same colour bars with different letters are significantly different *p*<0.05).

When ookinetes were incubated in RPMI supplemented with FBS at a density of 1 x10^7^ per ml, less than 20% had nuclei with condensed chromatin. Increasing the density of ookinetes to two or four x10^7^ per ml did not induce apoptosis, nor increase the number of parasites with compromised membranes (Figure 5A). Removal of FBS from the medium had no significant effect on the number of dead or dying ookinetes, suggesting that, in these conditions nutrients were adequate. Again no density effects were observed (Figure 5B). However, when incubated in PBS alone, the percentage of ookinetes with condensed chromatin in their nuclei increased to approximately 60%, although the number with compromised membranes remained below 20% (Figure 5C) and increasing incubation time to 24 hr resulted in over 90% of ookinetes exhibiting nuclei with condensed chromatin. Increasing ookinete density in these conditions still did not affect apoptosis or death, indeed similar results were observed at densities as high as eight and 16 x 10^7^ per ml in all three media (unpublished data). These data suggest that lack of nutrients may induce an apoptotic phenotype in *P. berghei* ookinetes but density had no effect.

## Discussion

The occurrence, semantics, function and evolutionary origin of cell death modalities in unicellular eukaryotes have been discussed at length [5,12,15,16,26,29] and will not be addressed further in this paper. Suffice to state that the detection of markers for apoptosis in protozoan parasites relies upon assays developed for metazoans, all of which have constraints associated with them [12] and none by themselves is conclusive [30]. Thus, where possible, several assays were used to corroborate observations in this work [31]. The investigation of chemical and ecological inducers of apoptosis found that the anti-malaria drug, CQ, caused a significant increase in the proportion of ookinetes with condensed chromatin, translocation of PS, caspase-like activity and DNA fragmentation, but did cause a loss of ΔΨ_m._ Staurosporine also induced chromatin condensation and caspase-like activity to increase without loss of ΔΨ_m._ and incubation with low doses of H_2_O_2_ resulted in a significant increase in two early markers of apoptosis, chromatin condensation and PS translocation; caspase-like activity and DNA fragmentation were not investigated. Finally, no effect of parasite density was observed, but withdrawal of nutrients also induced ookinetes to exhibit chromatin condensation although plasma membranes were intact. Although it is time consuming, cytofluorometry on a cell-by-cell basis is the most convenient method to detect features of apoptosis in the relatively small samples of ookinetes that could be collected for each experiment. Unfortunately, parasite yields and handling times precluded the use of all markers of apoptosis in every experiment, particularly those involving several chemical doses, parasite densities or incubation times.

The previous suggestion that exposure to low concentrations of H_2_O_2_ would induce markers of apoptosis was confirmed as the proportion of parasites displaying signs of chromatin condensation or PS translocation increased significantly at concentrations of 50 μM or below; whilst the plasma membrane remained intact. However, as shown previously [8], higher concentrations resulted in rapid death, detected as the proportion of PI positive ookinetes, with no detectable signs or features of apoptosis. Although death may have occurred by means other than apoptosis, a very rapid progression to late stage apoptosis at these higher concentrations cannot be ruled out, as discussed in [7].

The malaria ookinete is very susceptible to oxidative stress [32], as are the intra-erythrocytic stages. Of four effective selenium compounds tested against CQ-resistant *P. falciparum*, three generated ROS and caused a collapse of ΔΨ_m_ and DNA fragmentation, whereas one compound induced a similar apoptosis pathway without the production of ROS [21]. Unconjugated Bilirubin also induces oxidative stress in intra-erythrocytic stages and inhibits growth in culture, apparently because the parasites are undergoing apoptosis [20]. In that study, induction of oxidative stress was associated with loss of ΔΨ_m_ and up-regulation of *Pf*ARP expression, (a molecule that has the highest homology with TFAR-19, a mammalian TF-1 cell apoptosis-related gene). It also causes a six-fold activation of caspase-3-like proteases, which could be prevented with an inhibitor of caspase-3 activity, and condensation of nuclear chromatin was observed [20]. Kumar and colleagues [20] suggested that oxidative stress occurred as a result of Bilirubin-inhibited haemozoin formation, although other routes for the generation of ROS were not ruled out.

Ookinetes develop *in vivo* in a complex redox-active environment. It has been suggested that haem and haematin, released as the blood meal is digested, could react with iron and oxygen in the blood bolus to produce ROS [33]. Additionally, the midgut epithelial cells of infected mosquitoes are themselves a source of ROS (as discussed in [8]). Dual oxidase (Duox), a transmembrane protein present on the midgut epithelium, generates hydrogen peroxide and mediates an antimicrobial response to dietary bacteria and yeast in *Drosophila*[34]. However, it has recently been shown that in anopheline mosquitoes, Duox, together with immunomodulatory peroxidase (IMPer), makes the insect more susceptible to *Plasmodium* infection by forming a di-tyrosine network that inhibits activation of the epithelial immune system when bacteria or malaria parasites are present [35]. The role of epithelium-generated ROS in the induction of apoptosis in ookinetes in the midgut lumen is therefore uncertain.

In addition to extrinsic sources, it is possible that damaged mitochondria may themselves act as a major source of ROS that could initiate an apoptotic programmed. Indeed, ookinetes exhibiting loss of ΔΨ_m_ caused by a mitochondrial membrane potential disrupter were shown to display an apoptosis phenotype [7]. Loss of ΔΨ_m_ is also associated with the display of other markers of apoptosis, exhibited by an increasing proportion of ookinetes as the length of time they are incubated in PBS or ookinete medium increases [8,23]. These findings point to a role for mitochondria in a putative apoptosis pathway in *P. berghei* ookinetes, however, findings presented here, and elsewhere, suggest that an amitochondrial pathway also exists [8].

The reports that CQ causes a significant decline in the proportion of *P. falciparum* trophozoites with functional ΔΨ_m_[9,10] are at odds with these findings for *P. berghei* ookinetes. Despite finding a significant increase in the proportion of parasites with condensed chromatin, caspase-like activity, PS translocation and fragmented DNA, loss of ΔΨ_m_ following a 2-h incubation with 1 mM CQ could not be detected. Interestingly, Srivastava et al. [36] found that CQ was unable to disrupt the mitochondrial membrane potential of another species of rodent malaria, *Plasmodium yoelii,* at concentrations up to 10^-3^ M, whereas the broad spectrum anti-malarial drug, Atovaquone, caused loss of ΔΨ_m_ at far lower doses.

Similar to findings for CQ exposure, ookinetes incubated with ST did not loose ΔΨ_m_, although there was a significant increase in the proportion with condensed chromatin and activated cysteine proteases. This is also in contrast to its effect on trophozoites, where it did cause loss of ΔΨ_m_[10]. In the latter study, ST treatment significantly increased CaspaTag binding, but this was not inhibited by the cysteine protease inhibitor zVAD, although binding induced by CQ was. This suggests that different cysteine proteases may be involved in the pathways induced by these drugs in *P. falciparum* trophozoites and in *P. berghei*, but pathways in ookinetes clearly need more investigation before any conclusions can be drawn.

One possible explanation for the apparent differences in the role of mitochondria in CQ-induced apoptosis in malaria parasites is the timing of exposure to CQ. Ch’ng and co-workers [10] incubated trophozoites for 4–6 hr before detecting apoptosis markers. Monitoring this length of drug exposure is difficult as, when maintained in ookinete medium, the proportion of ookinetes that display features of apoptosis increases with time, even when no apoptosis-inducer is added to the medium [6,7]. However, as several markers of apoptosis increase significantly following just 2-hr exposure to CQ, these differences are not attributed to the need for longer exposure time. It is likely that CQ induces apoptosis via a pathway that does not involve mitochondria in ookinetes. As the pathway for the action of CQ that Ch’ng et al. propose [10] could not occur in ookinetes, due to the absence of a digestive vacuole, the drug may initially accumulate somewhere else, act directly in the cytoplasm to activate cysteine proteases, or work via another route. Chloroquine is known to be cytotoxic to mammalian cells and molecular mechanisms including MOMP, the induction of NO synthesis, glutathione depletion and cellular redox changes have been implicated [37,38]. Of note here is the observation that, at the specific time investigated, more ookinetes have condensed chromatin than caspase-like activity and fewer still had fragmented DNA. This may have some bearing on the timing of the pathway by which the apoptotic phenotypes are manifested. The elucidation of apoptotic pathways occurring in ookinetes will also shed light on CQ action.

The finding that micromolar concentrations of CQ induce ookinetes to undergo apoptosis could have implications for their survival *in vivo*. The effect of the administration of CQ to vertebrate hosts on malaria transmission has been widely investigated in the laboratory and the field. Conflicting findings have been reported, but enhanced transmission has often been associated with CQ-resistant *Plasmodium* compared to susceptible ones and it has been suggested that selection for these parasites may be due to increased survival of gametocytes or sporogonic stages when under CQ pressure (reviewed in [39]). However, it is probable that blood meals taken following CQ treatment may not contain sufficiently high concentrations of CQ to induce apoptosis in zygotes and ookinetes. In the study reported herein, 416 μM CQ was the lowest concentration that induced a significant increase in apoptotic ookinetes. This is considerably higher than the treatment with nanomolar concentrations of CQ that causes accumulation in the digestive vacuole, alteration of haemozoin polymerization and trophozoite killing, and even the 3 μM concentration that induces permeabilization of the digestive vacuole and triggers apoptosis, as discussed by Ch’ng and colleagues [40]. These authors reviewed various clinical trials involving dose escalation that may have resulted in micromolar levels of CQ in the blood. They also discuss the feasibility of redeploying CQ, using regimes/formulations that that may induce programmed cell death in the parasite [40]. The likelihood of such regimes producing sustained blood concentrations high enough to be active upon mosquito stages of *Plasmodium* is remote but, as an apoptosis inducer that is active *in vitro,* CQ has a role to play in future investigations of the pathways involved in apoptosis in ookinetes.

The apoptosis triggers discussed so far result from a toxic external environment that may, or may not, be influenced by the presence of other ookinetes. Several authors have proposed that protozoan parasites may undergo apoptosis in a density dependent manner [26,41]. Reece and colleagues [4], review previous proposals that the removal, by suicide, of a proportion of the population could benefit the remaining parasites by preventing premature host death, by avoiding the stimulation of a density related immune response or removal of less fit members [42,43]; all of these scenarios are density dependent. Studies of the population dynamics of early sporogony have demonstrated losses at all transitional stages within the mosquito midgut (reviewed in [44]) and recently Sinden and colleagues found negative density-dependence operating for densities greater than 355 ookinetes per *Anopheles stephensi*[45] and speculated that factors such as a density-dependent, parasite-killing mechanism or competition for resources may be operating in the mosquito. The ookinete densities used here (starting at 10,000 per μl) were far greater than those calculated to initiate a density dependent effect *in vivo*[45]. Although no density-dependent induction of apoptosis was demonstrated, this might be due to factors associated with experimental design such as the developmental stage investigated (for example, signaling molecules produced earlier may have been removed during ookinete harvesting) or the limited period of incubation at high density. In view of the proposed function of density-driven apoptosis as a method of improving the fitness of a clonal population of ookinetes, further investigation using different experimental approaches, additional assays for apoptosis and, in particular, experiments *in vivo*, need to be performed before density can be completely ruled out as a trigger for apoptosis*.*

Clearly nutrients are not limiting when incubation is in RPMI, but incubation for 4 hr in PBS alone resulted in a large increase in ookinetes with condensed chromatin, although no density effect was noted. As multiple markers for apoptosis were not examined for this experiment, the conclusion that nutrient depletion resulted in an apoptotic phenotype rather than autophagy should be viewed with caution.

## Conclusion

These, and previous results [8], strongly suggest that ookinetes respond to adverse conditions such as redox stress or drug treatment; orchestrating their own death and exhibiting a phenotype akin to metazoan apoptosis. Multiple mechanisms may exist as there appears to be both a mitochondrial and an amitochondrial pathway involved, and pathways may differ between erythrocytic stages and ookinetes, or between rodent and human malaria parasites. So far, there is more experimental evidence to support the view that apoptosis is a way to die when subjected to factors that may be of host origin, rather than a strategy employed by ookinetes to increase the fitness of surviving parasites (discussed in [3]). However, further investigations are required, particularly *in vivo* and with mixed clones of *Plasmodium*, before a direct role for density-dependent mechanisms is dismissed, as relatedness is also essential if apoptosis is to be regarded as a beneficial trait [4]. This apoptotic trait has probabilistic expression as a proportion of ookinetes survive the expression of apoptotic triggers; understanding what governs this, and the pathways operating in *Plasmodium* ookinetes may inform novel transmission-blocking strategies if the knowledge can be used to cause all ookinetes to commit suicide in the midgut lumen.

## Competing interests

The authors declare that they have no competing interests

## Authors' contributions

HM, MA, JH and HS performed the experiments and statistical analysis. VC and AU assisted with experiments. HH conceived the study and drafted the manuscript. HM, MA, JH and HS assisted with the study design and drafting of the manuscript and all authors read and approved the final manuscript.

## References

[B1] Picot S Burnod J Bracchi V Chumpitazi BF Ambroise-Thomas P Apoptosis related to chloroquine sensitivity of the human malaria parasite *Plasmodium falciparum* Trans R Soc Trop Med Hyg 1997 91 590 591 10.1016/S0035-9203(97)90039-0 9463676

[B2] Hurd H Carter V Nacer A Interactions between malaria and mosquitoes: the role of apoptosis in parasite establishment and vector response to infection Curr Top Microbiol Immunol 2005 289 185 217 10.1007/3-540-27320-4_9 15791957

[B3] Pollitt LC Colegrave N Khan SM Sajid M Reece SE Investigating the evolution of apoptosis in malaria parasites: the importance of ecology Parasit Vectors 2010 3 105 10.1186/1756-3305-3-105 21080937 PMC3136143

[B4] Reece SE Pollitt LC Colegrave N Gardner A The meaning of death: evolution and ecology of apoptosis in protozoan parasites PLoS Pathog 2011 7 e1002320 10.1371/journal.ppat.1002320 22174671 PMC3234211

[B5] Engelbrecht D Durand PM Coetzer TL On programmed cell death in *Plasmodium falciparum*: status quo J Trop Med 2012 2012 646534 22287973 10.1155/2012/646534 PMC3263642

[B6] Al-Olayan EM Williams GT Hurd H Apoptosis in the malaria protozoan, *Plasmodium berghei*: a possible mechanism for limiting intensity of infection in the mosquito Int J Parasitol 2002 32 1133 1143 10.1016/S0020-7519(02)00087-5 12117496

[B7] Arambage SC Grant KM Pardo I Ranford-Cartwright L Hurd H Malaria ookinetes exhibit multiple markers for apoptosis-like programmed cell death *in vitro* Parasit Vectors 2009 2 32 10.1186/1756-3305-2-32 19604379 PMC2720949

[B8] Ali M Al-Olayan EM Lewis S Matthews H Hurd H Naturally occurring triggers that induce apoptosis-like programmed cell death in *Plasmodium berghei* ookinetes PLoS One 2010 5 12634 10.1371/journal.pone.0012634 PMC2936559 20844583

[B9] Meslin B Barnadas C Boni V Latour C De Monbrison F Kaiser K Picot S Features of apoptosis in *Plasmodium falciparum* erythrocytic stage through a putative role of PfMCA1 metacaspase-like protein J Infect Dis 2007 195 1852 1859 10.1086/518253 17492602

[B10] Ch'ng JH Kotturi SR Chong AG Lear MJ Tan KS A programmed cell death pathway in the malaria parasite *Plasmodium falciparum* has general features of mammalian apoptosis but is mediated by clan CA cysteine proteases Cell Death Dis 2010 1 e26 10.1038/cddis.2010.2 21364634 PMC3032337

[B11] Mutai BK Waitumbi JN Apoptosis stalks *Plasmodium falciparum* maintained in continuous culture condition Malar J 2010 9 Suppl 3 S6 10.1186/1475-2875-9-S3-S6 21144086 PMC3002142

[B12] Jimenez-Ruiz A Alzate JF MacLeod ET Luder CGK Fasel N Hurd H Apoptotic markers in protozoan parasites Parasit Vectors 2010 3 104 10.1186/1756-3305-3-104 21062457 PMC2993696

[B13] Meslin B Beavogui AH Fasel N Picot S *Plasmodium falciparum* metacaspase PfMCA-1 triggers a z-VAD-fmk inhibitable protease to promote cell death PLoS One 2011 6 e23867 10.1371/journal.pone.0023867 21858231 PMC3157471

[B14] Meslin B Zalila H Fasel N Picot S Bienvenu AL Are protozoan metacaspases potential parasite killers? Parasit Vectors 2011 4 26 10.1186/1756-3305-4-26 21356053 PMC3058108

[B15] Nedelcu AM Comparative genomics of phylogenetically diverse unicellular Eukaryotes provide new insights into the genetic basis for the evolution of the programmed cell death machinery J MolEvol 2009 68 256 268 10.1007/s00239-009-9201-1 19209377

[B16] Kaczanowski S Sajid M Reece SE Evolution of apoptosis-like programmed cell death in unicellular protozoan parasites Parasit Vects 2011 4 44 10.1186/1756-3305-4-44 PMC3077326 21439063

[B17] Guha M Choubey V Maity P Kumar S Shrivastava K Puri SK Bandyopadhyay U Overexpression, purification and localization of apoptosis related protein from *Plasmodium falciparum* Protein Expr Purif 2007 52 363 372 10.1016/j.pep.2006.11.004 17182255

[B18] Rathore S Jain S Sinha D Gupta M Asad M Srivastava A Narayanan MS Ramasamy G Chauhan VS Gupta D Mohmmed A Disruption of a mitochondrial protease machinery in *Plasmodium falciparum* is an intrinsic signal for parasite cell death Cell Death Dis 2011 2 e231 10.1038/cddis.2011.118 22113196 PMC3223699

[B19] Sharma N Mohanakrishnan D Shard A Sharma A Sinha AK Sahal D Saima Stilbene-chalcone hybrids: design, synthesis, and evaluation as a new class of antimalarial scaffolds that trigger cell death through stage specific apoptosis J Med Chem 2012 55 297 311 22098429 10.1021/jm201216y

[B20] Kumar S Guha M Choubey V Maity P Srivastava K Puri SK Bandyopadhyay U Bilirubin inhibits *Plasmodium falciparum* growth through the generation of reactive oxygen species Free Radic Biol Med 2008 44 602 613 10.1016/j.freeradbiomed.2007.10.057 18070610

[B21] Suradji EW Hatabu T Kobayashi K Yamazaki C Abdulah R Nakazawa M Nakajima-Shimada J Koyama H Selenium-induced apoptosis-like cell death in *Plasmodium falciparum* Parasitology 2011 138 1 11 21854677 10.1017/S0031182011001399

[B22] Ch'ng JH Liew K Goh AS Sidhartha E Tan KS Drug-induced permeabilization of parasite's digestive vacuole is a key trigger of programmed cell death in *Plasmodium falciparum* Cell Death Dis 2011 2 e216 10.1038/cddis.2011.97 21993392 PMC3219093

[B23] Arambage SC An in vitro study of apoptosis-like cell death in Plasmodium berghei 2008 Institute for Science and Technology in Medicine: Keele University

[B24] James ER Green DR Manipulation of apoptosis in the host-parasite interaction Trends Parasitol 2004 20 280 287 10.1016/j.pt.2004.04.004 15147679

[B25] Mideo N Reece SE Plasticity in parasite phenotypes: evolutionary and ecological implications for disease Future Microbiol 2012 7 17 24 10.2217/fmb.11.134 22191443

[B26] Hurd H Carter V The role of programmed cell death in *Plasmodium*-mosquito interactions Int J Parasitol 2004 34 1459 1472 10.1016/j.ijpara.2004.10.002 15582523

[B27] Carter V Nacer AM Underhill A Sinden RE Hurd H Minimum requirements for ookinete to oocyst transformation in *Plasmodium* Int J Parasitol 2007 37 1221 1232 10.1016/j.ijpara.2007.03.005 17482621 PMC2474741

[B28] Salvioli S Ardizzoni A Franceschi C Cossarizza A JC-1, but not DiOC(6)(3) or rhodamine 123, is a reliable fluorescent probe to assess Delta Psi changes in intact cells: Implications for studies on mitochondrial functionality during apoptosis FEBS Lett 1997 411 77 82 10.1016/S0014-5793(97)00669-8 9247146

[B29] van Zandbergen G Luder CGK Heussler V Duszenko M Programmed cell death in unicellular parasites: a prerequisite for sustained infection? Trends Parasitol 2010 26 477 483 10.1016/j.pt.2010.06.008 20591738

[B30] Kroemer G Galluzzi L Vandenabeele P Abrams J Alnemri ES Baehrecke EH Blagosklonny MV El-Deiry WS Golstein P Green DR Hengartner M Knight RA Kumar S Lipton SA Malorni W Nuňez G Peter ME Tschopp J Yuan J Piacentini M Zhivotovsky B Melino G Classification of cell death: recommendations of the Nomenclature Committee on Cell Death 2009 Cell Death Differ 2009 16 3 11 10.1038/cdd.2008.150 18846107 PMC2744427

[B31] Galluzzi L Aaronson SA Abrams J Alnemri ES Andrews DW Baehrecke EH Bazan NG Blagosklonny MV Blomgren K Borner C Bredesen DE Brenner C Castedo M Cidlowski JA Ciechanover A Cohen GM De Laurenzi V De Maria R Deshmukh M Dynlacht BD El-Deiry WS Flavell RA Fulda S Garrido C Golstein P Gougeon ML Green DR Gronemeyer H Hajnoczky G Hardwick JM Hengartner MO Ichijo H Jaattela M Kepp O Kimchi A Klionsky DJ Knight RA Kornbluth S Kumar S Levine B Lipton SA Lugli E Madeo F Malorni W Marine JCW Martin SJ Medema JP Mehlen P Melino G Moll UM Morselli E Nagata S Nicholson DW Nicotera P Nunez G Oren M Penninger J Pervaiz S Peter ME Piacentini M Prehn JHM Puthalakath H Rabinovich GA Rizzuto R Rodrigues CMP Rubinsztein DC Rudel T Scorrano L Simon HU Steller H Tschopp J Tsujimoto Y Vandenabeele P Vitale I Vousden KH Youle RJ Yuan J Zhivotovsky B Kroemer G Guidelines for the use and interpretation of assays for monitoring cell death in highereukaryotes Cell Death Differ 2009 16 1093 1107 10.1038/cdd.2009.44 19373242 PMC2757140

[B32] Lanz-Mendoza H Hernandez-Martinez S Ku-Lopez M Rodriguez Mdel C Herrera-Ortiz A Rodriguez MH Superoxide anion in *Anopheles albimanus* hemolymph and midgut is toxic to *Plasmodium berghei* ookinetes J Parasitol 2002 88 702 706 12197117 10.1645/0022-3395(2002)088[0702:SAIAAH]2.0.CO;2

[B33] Peterson TM Gow AJ Luckhart S Nitric oxide metabolites induced in *Anopheles stephensi* control malaria parasite infection Free Radic Biol Med 2007 42 132 142 10.1016/j.freeradbiomed.2006.10.037 17157200 PMC1764505

[B34] Ha EM Oh CT Bae YS Lee WJ A direct role for dual oxidase in *Drosophila* gut immunity Science 2005 310 847 850 10.1126/science.1117311 16272120

[B35] Kumar S Molina-Cruz A Gupta L Rodrigues J Barillas-Mury C A peroxidase/dual oxidase system modulates midgut epithelial immunity in *Anopheles gambiae* Science 2010 327 1644 1648 10.1126/science.1184008 20223948 PMC3510679

[B36] Srivastava IK Rottenberg H Vaidya AB Atovaquone, a broad spectrum antiparasitic drug, collapses mitochondrial membrane potential in a malarial parasite J Biol Chem 1997 272 3961 3966 10.1074/jbc.272.7.3961 9020100

[B37] Park BC Park SH Paek SH Park SY Kwak MK Choi HG Yong CS Yoo BK Kim JA Chloroquine-induced nitric oxide increase and cell death is dependent on cellular GSH depletion in A172 human glioblastoma cells Toxicol Lett 2008 178 52 60 10.1016/j.toxlet.2008.02.003 18359172

[B38] Jiang PD Zhao YL Shi W Deng XQ Xie G Mao YQ Li ZG Zheng YZ Yang SY Wei YQ Cell growth inhibition, G2/M cell cycle arrest, and apoptosis induced by chloroquine in human breast cancer cell line Bcap-37 Cell Physiol Biochem 2008 22 431 440 10.1159/000185488 19088425

[B39] Butcher GA Antimalarial drugs and the mosquito transmission of *Plasmodium* Int J Parasitol 1997 27 975 987 10.1016/S0020-7519(97)00079-9 9363480

[B40] Ch'ng JH Renia L Nosten F Tan KS Can we teach an old drug new tricks? Trends Parasitol 2012 28 220 224 10.1016/j.pt.2012.02.005 22445323

[B41] Welburn SC Maudlin I Control of *Trypanosoma brucei brucei* infections in tsetse, *Glossina morsitans* Med Vet Entomol 1997 11 286 289 10.1111/j.1365-2915.1997.tb00408.x 9330261

[B42] Duszenko M Figarella K Macleod ET Welburn SC Death of a trypanosome: a selfish altruism Trends Parasitol 2006 22 536 542 10.1016/j.pt.2006.08.010 16942915

[B43] Hurd H Grant KM Arambage SC Apoptosis-like death as a feature of malaria infection in mosquitoes Parasitology 2006 132 Suppl S33 47 17018164 10.1017/S0031182006000849

[B44] Vaughan JA Population dynamics of *Plasmodium* sporogony Trends Parasitol 2007 23 63 70 10.1016/j.pt.2006.12.009 17188574

[B45] Sinden RE Dawes EJ Alavi Y Waldock J Finney O Mendoza J Butcher GA Andrews L Hill AV Gilbert SC Basanez MG Progression of *Plasmodium berghei* through *Anopheles stephensi* is density-dependent PLoS Pathog 2007 3 e195 10.1371/journal.ppat.0030195 18166078 PMC2156095

